# Role of Syndiniales parasites in depth-specific networks and carbon flux in the oligotrophic ocean

**DOI:** 10.1093/ismeco/ycae014

**Published:** 2024-01-23

**Authors:** Sean R Anderson, Leocadio Blanco-Bercial, Craig A Carlson, Elizabeth L Harvey

**Affiliations:** Department of Biological Sciences, University of New Hampshire, Durham, NH 03824, United States; Marine Chemistry and Geochemistry Department, Woods Hole Oceanographic Institution, Falmouth, MA 02543, United States; Bermuda Institute of Ocean Sciences, Arizona State University, St. George’s GE 01, Bermuda; Department of Ecology, Evolution and Marine Biology and the Marine Science Institute, University of California, Santa Barbara, CA 93106, United States; Department of Biological Sciences, University of New Hampshire, Durham, NH 03824, United States

**Keywords:** syndiniales, parasitism, plankton ecology, species networks, amplicon metabarcoding, POC flux, Sargasso Sea

## Abstract

Microbial associations that result in phytoplankton mortality are important for carbon transport in the ocean. This includes parasitism, which in microbial food webs is dominated by the marine alveolate group, Syndiniales. Parasites are expected to contribute to carbon recycling via host lysis; however, knowledge on host dynamics and correlation to carbon export remain unclear and limit the inclusion of parasitism in biogeochemical models. We analyzed a 4-year 18S rRNA gene metabarcoding dataset (2016–19), performing network analysis for 12 discrete depths (1–1000 m) to determine Syndiniales–host associations in the seasonally oligotrophic Sargasso Sea. Analogous water column and sediment trap data were included to define environmental drivers of Syndiniales and their correlation with particulate carbon flux (150 m). Syndiniales accounted for 48–74% of network edges, most often associated with Dinophyceae and Arthropoda (mainly copepods) at the surface and Rhizaria (Polycystinea, Acantharea, and RAD-B) in the aphotic zone. Syndiniales were the only eukaryote group to be significantly (and negatively) correlated with particulate carbon flux, indicating their contribution to flux attenuation via remineralization. Examination of Syndiniales amplicons revealed a range of depth patterns, including specific ecological niches and vertical connection among a subset (19%) of the community, the latter implying sinking of parasites (infected hosts or spores) on particles. Our findings elevate the critical role of Syndiniales in marine microbial systems and reveal their potential use as biomarkers for carbon export.

## Introduction

Phytoplankton are central to the biological carbon pump, fixing atmospheric carbon dioxide and contributing to the sinking of organic matter [1]. The biological carbon pump exports ~10 Pg C y^−1^ from the surface oceans, with contributions from many biological, chemical, and physical processes [2, 3]. Particulate organic carbon (POC) fixed by primary producers is exported via several well-known pathways: gravitational sinking, aggregation, active transport from diel migrators, and physical mixing [4–6]. Phytoplankton mortality, and the species interactions that underpin it, also drive export strength and efficiency [7, 8]. However, mechanistic links between species interactions and carbon export remain elusive and preclude incorporation of mortality in ocean carbon export models [9, 10]. Insight into microbial interactions is necessary to define the contribution of plankton mortality to carbon cycling and accurately resolve the ecological mechanisms that drive carbon export.

Parasitism is arguably the most common lifestyle employed by organisms on Earth [11], and yet in the ocean, it is rarely incorporated in food web or biogeochemical models [12]. Routine metabarcoding surveys have revealed the hidden diversity and global distribution of protist parasites in the ocean [13, 14]. Marine alveolates in the group Syndiniales are the most ubiquitous and phylogenetically diverse, with sequences observed in virtually all marine biomes [15–17]. Syndiniales are represented by five main groups (Groups I–V), with Group II being the most diverse and well-studied, particularly members of the genus *Amoebophyra* that are known to infect dinoflagellates [15]. In addition to pelagic samples, several recent studies have reported a high proportion of Syndiniales reads in particles collected from sediment traps [10, 18–21]. Yet, direct links between Syndiniales and carbon export have not been established, making it difficult to contextualize the role of parasites in carbon cycling.

Carbon exported to the deep ocean is partly mediated by organismal interactions [7, 22], which, for parasites, have not been well-defined vertically in the water column [23]. Syndiniales infect a wide range of hosts (e.g., dinoflagellates, ciliates, radiolarians, and copepods), with several studies reporting top-down pressure on coastal phytoplankton blooms that rival grazing loss [15, 24]. Infection begins with an attachment of a motile parasite spore (<10 μm) to a host cell, followed by rapid digestion of host material and growth inside the nucleus [25–27]. After 2–3 days, whereby the parasite increases in volume up to 200-fold, the host ruptures and releases hundreds of new spores into the environment [27]. It is estimated that up to 70% of host biomass is released as labile dissolved organic matter (DOM) that can be recycled by heterotrophic bacteria [28]. Estimates of parasite-released DOM, and its lability, likely depend on host community composition and biomass [29], as well as environmental conditions, such as nutrient concentrations and temperature [30, 31]. Nevertheless, building evidence suggests that Syndiniales are central to microbial food webs and carbon cycling, making it imperative to detail host associations with depth and their involvement in carbon export.

Here, we investigate Syndiniales infection dynamics throughout the photic (1–120 m) and aphotic (160–1000 m) zones at the Bermuda Atlantic Time-series Study (BATS) site, a long-term (>30 years) ocean monitoring program in the seasonally oligotrophic Sargasso Sea [32]. Recent 18S rRNA gene metabarcoding work at BATS revealed a high relative abundance of Syndiniales (~40%) throughout the water column [33] and their presence in sediment traps [20]. Moreover, several studies at BATS [33, 34], and in other oligotrophic systems [17], have observed significant shifts in protist diversity and composition with depth, with communities varying less over seasonal cycles. We performed covariance network analysis of a 4-year (2016–19) 18S rRNA gene metabarcoding dataset [33], constructing networks for each of 12 discrete depths (1–1000 m). Our analysis revealed potential Syndiniales–host pairings that varied with increasing depth. Sediment trap data collected from the same location revealed a significant and negative correlation between Syndiniales and bulk POC flux at 150 m, implying that increased parasite relative abundance may enhance flux attenuation through remineralization of host carbon. In addition, we found evidence of vertical transport among a subset (19%) of the parasite community, matching the percent of primary production (10–20%) exported from the surface oceans each year.

## Results and Discussion

### Spatiotemporal variability and environmental drivers of Syndiniales

**Figure 1:**
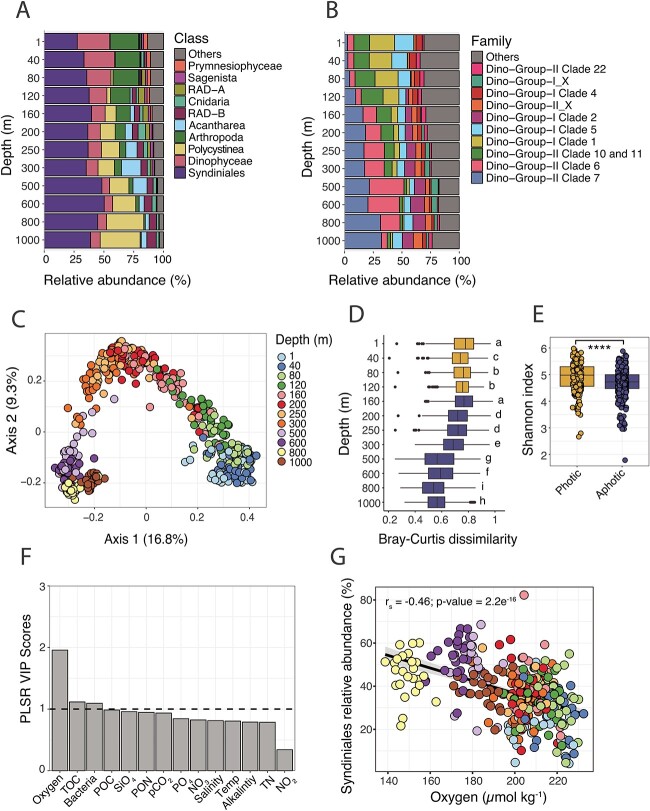
Syndiniales population dynamics and environmental drivers at BATS. Stacked bar plots of 18S relative abundance at the class level (**A**) and within Syndiniales (**B**) at the family (clade) level at each depth. Stacked bar plots display the top 10 groups based on relative abundance (‘others’ in gray). All Syndiniales ASVs were considered for (**B**). Values are presented as the mean at each depth and across all time points; *n* = 43–53. (**C**) Principal coordinates analysis based on Bray–Curtis dissimilarity of all samples, filtered to only include Syndiniales ASVs. Samples represent single replicates. The proportion of variance explained by the first two axes is indicated. (**D**) Bray–Curtis dissimilarity as a function of sampling depth. Values represent the mean ± SD (*n* = 43–53), with letters indicating statistical differences. Samples are represented by their position in the photic (gold) vs. aphotic zones (blue). (**E**) Shannon index values for Syndiniales ASVs in the photic (*n* = 206) vs. aphotic zones (*n* = 381). A Wilcoxon test revealed significant differences in the mean (^*^^*^^*^^*^ = *P*-value <0.0001). (**F**) VIP scores for each factor estimated from the PLSR model and ordered from highest to lowest. VIP > 1 (dashed line) indicate variables most important to the model. Oxygen (μmol kg^−1^); TOC = total organic carbon (μmol kg^−1^); bacteria = bacteria cell density (10^8^ cells kg^−1^); POC = particulate organic carbon (μg kg^−1^); SiO_4_ = silicate (μmol kg^−1^); PON = particulate organic nitrogen (μg kg^−1^); *p*CO_2_ = partial pressure of carbon dioxide (μatm); PO_4_ = phosphate (μmol kg^−1^); NO_3_ = nitrate (μmol kg^−1^); salinity (psu); temp = temperature (°C); alkalinity (μmol kg^−1^); TN = total nitrogen (μmol kg^−1^); NO_2_ = nitrite (μmol kg^−1^). (**G**) Spearman rank correlation (*r_s_*) between Syndiniales relative abundance and oxygen concentration. All samples at all depths were considered. Regression line and 95% confidence intervals are shown.

A total of 18 643 unique Syndiniales amplicon sequence variants (ASVs) were identified over the entire 18S dataset, which included monthly whole water samples (>0.22 μm) collected over 4 years (2016–19) and 12 discrete depths (1–1000 m). Syndiniales were present with depth and over time [33], accounting for 26–59% of total 18S reads (Fig. 1A; Supplementary Fig. S1). Other studies in oligotrophic systems have found that Syndiniales comprise a significant proportion of the eukaryotic population in deep waters [17, 35, 36] and sediment traps [18, 37]. By comparison, relative abundance of other major 18S groups were more variable with depth [33], with higher Dinophyceae and Arthropoda (mainly copepods) relative abundance in the photic zone that shifted to a Rhizaria-dominated community in the aphotic zone and included several radiolarians such as Polycystinea, Acantharea, and RAD-B (Fig. 1A; Supplementary Fig. S1).

Syndiniales were largely assigned to Dino-Groups I and II (Supplementary Fig. S2), which are considered the two most prevalent and diverse Syndiniales groups in the ocean [15]. Depth variability was most apparent at the family (clade) level (Fig. 1B). Several clades, such as Dino-Group-II Clade 10 + 11 and Dino-Group-I Clades 1 and 5, had higher relative abundance in the photic zone, while Dino-Group-II Clades 6 and 7 and Dino-Group-I Clade 2 were more prevalent in the aphotic zone (Fig. 1B). Clade-level patterns observed at BATS align closely with previous observations of Syndiniales distribution in the water column [15, 37], further supporting the presence of depth-dependent ecological niches within Syndiniales. As is the case with metabarcoding data, the relative abundance of many eukaryotic groups, including Syndiniales, may be inflated due to high 18S gene copy number [38]. We focused our analysis on depth-related trends within Syndiniales and note that other groups at the surface (e.g., dinoflagellates) were also biased by potential copy number.

Syndiniales community composition at BATS was significantly influenced by depth (PERMANOVA *R*^2^ = 0.38; *P*-value < 0.001), with communities being less diverse and having lower dissimilarity values on average (higher within-group similarity) in the aphotic zone (Fig. 1C–E). Syndiniales composition was significantly different (*P*-value < 0.01) between most discrete sampling depths, except for 1 vs. 40 m and 250 vs. 300 m (Table S1). Though significant, Syndiniales composition over the entire dataset was only weakly clustered by season (PERMANOVA *R*^2^ = 0.02; *P*-value < 0.001; Table S1). As in [33], we observed seasonal variability when analyzing single depths (Supplementary Fig. S3). For example, Syndiniales composition varied by season (PERMANOVA *R*^2^ = 0.2 and 0.11; *P*-value < 0.001) at depths in the photic zone (e.g., 1 and 120 m), while seasonal changes were not significant at deeper depths (e.g., 600 and 1000 m). Other 18S rRNA gene metabarcoding studies at this site [33, 34] and in the North Pacific [17] have noted significant variability in protist composition and diversity with depth compared with weaker seasonal signals. Seasonality in the Sargasso Sea is characterized by deep convective mixing (~150–300 m) between January and March and stratification of the water column (mixed layer depth < 20 m) as early as May [32], driving seasonal patterns in phytoplankton, heterotrophic bacteria, and viruses [39–41]. Enhanced winter mixing typically facilitates higher plankton biomass, net primary production, and carbon export to 150–300 m [32, 42]. Seasonal effects on Syndiniales may be minimized in the mixed layer due to their ubiquity, wide host range, and tolerance for variable physical and chemical conditions. Others have noted seasonal patterns among Syndiniales in coastal time series with weekly resolution [43–45]. Sampling intervals were less resolved (monthly) at BATS, which may have masked temporal effects.

Partial least squares regression (PLSR) was used to identify the most important environmental variables that influenced rarefied Syndiniales read counts at BATS (Fig. 1F). Vertical profiles of hydrographic data, nutrient concentrations, and carbon measurements (Supplementary Fig. S4; Table S2) were typical for this site [42]. Oxygen concentration was most important in explaining Syndiniales read counts (Fig. 1F) based on variable importance on the projection (VIP) scores from the PLSR model (Supplementary Fig. S5). Other variables were also important (VIP > 1), including total organic carbon (TOC) concentration and bacterioplankton cell density (Fig. 1F). Syndiniales relative abundance was negatively correlated with oxygen concentration (Spearman *r_s_* = −0.46; *P*-value < 0.001), with highest abundance in the aphotic zone where oxygen was <180 μmol kg^−1^ (Fig. 1G). Syndiniales are known to persist and contribute to species interactions in low-oxygen environments [23, 46, 47] and other extreme marine habitats, such as hydrothermal vents [48]. For instance, Syndiniales accounted for 11–99% of 18S reads (0.2–1.6 μm) in anoxic waters off the northern coast of Chile, implying parasites were largely present as spores and likely infecting resident hosts [47]. Protists, such as Syndiniales, may have adapted strategies to function under oxygen-limited conditions [49]. It will be important to closely examine the influence of oxygen on protist parasites in the field and through culture-based work, including how oxygen concentrations may affect host viability and infectivity.

### Depth-specific networks and putative parasite–host relationships

Co-occurrence networks are often applied to amplicon sequencing data to infer species relationships in the ocean [31, 50]. Network analysis is a powerful, hypothesis-driven approach, particularly as many marine microbial species remain uncultured and have unrealized ecological functions [51, 52]. Caution should be used when interpreting network results, as correlations cannot be defined as true ecological interactions [53]. Syndiniales were present in high relative abundance in the photic and aphotic zones at BATS and were significantly influenced by sampling depth. Therefore, we focused our network analysis on identifying prospective Syndiniales–host relationships at different depths in the water column, an important step toward defining enigmatic infection dynamics in the context of carbon export. Networks were constructed at each of 12 discrete depths (1–1000 m) using SPIEC-EASI, a program designed to infer direct (and statistically significant) associations between ASVs, while minimizing false connections that often arise from compositional amplicon datasets [54]. To further minimize dense networks [51], only the top 150 most abundant 18S ASVs were selected and included in each depth network, which closely represented the 18S community as a whole and the major Syndiniales clades at each depth (Supplementary Fig. S6).

**Figure 2:**
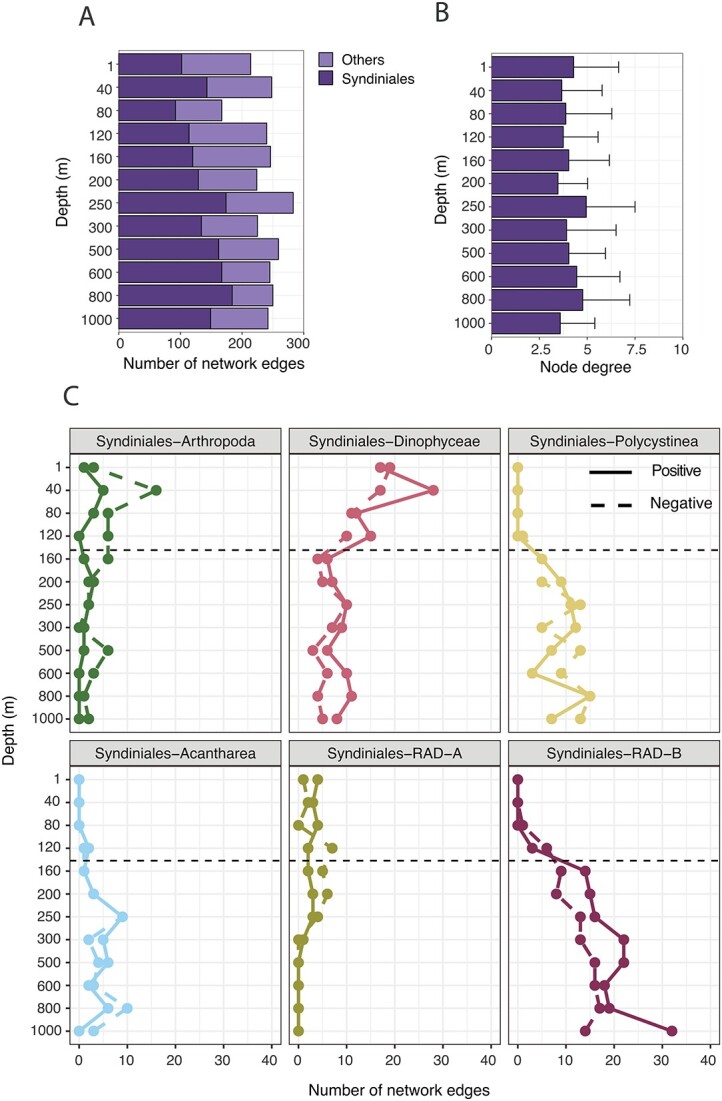
Results from SPIEC-EASI network analysis that considered the top 150 18S ASVs at each depth. (**A**) The number of network edges (associations) attributed to Syndiniales ASVs (dark purple) and those connected to other eukaryotes (light purple). The sum of both represents the total number of edges at each depth. (**B**) Syndiniales node degree for each depth network, i.e., the number of other 18S ASVs connected to a single Syndiniales node (or ASV). Values represent the mean ± SD at each depth. (**C**) Depth-related trends display the number of positive (solid) and negative (dashed) edges between Syndiniales and putative host groups at the class level (Arthropoda, Dinophyceae, Polycystinea, Acantharea, and RAD-A/B). The transition from photic to aphotic zones are distinguished with dashed lines at 140 m (0.1% light). Missing data points indicate that specific relationships were not detected at that depth. Network edges filtered for Syndiniales are in Table S3.

Depth networks at BATS were dominated by Syndiniales (Fig. 2A). A total of 268 Syndiniales ASVs accounted for 48–74% of 18S edges across networks, with a slight increase in edge number with increasing depth (Fig. 2A). Other sequence-based studies have noted a large contribution of Syndiniales ASVs to species edges inferred by co-occurrence networks [45, 50, 55]. Within networks, a single Syndiniales ASV was often connected to more than two putative host ASVs on average, with little variation with depth (Fig. 2B). Similar patterns were observed for potential host ASVs connected to different parasites (Supplementary Fig. S7), with node degree often >2. However, node degree of potential host groups was vertically structured at times, especially for Arthropoda, with a peak in node degree at 120–200 m (Supplementary Fig. S7). These findings, along with evidence from other sequencing [15, 56] and microscopy-based analyses [30], point to Syndiniales infecting a wide host range. Though this may not always be the case, as infection appears to be highly specific for certain parasite–host strains [57], especially members of the genus *Amoebophyra* that infect harmful bloom-forming dinoflagellates in coastal and eutrophic regions [58]. Additional quantitative work is required to confirm infection patterns under varying conditions and in different habitats [52].

Syndiniales were most often associated with several eukaryotic groups, such as Arthropoda, Dinophyceae, and Rhizaria (Polycystinea, Acantharea, and RAD-A/B), all representing verified or potential hosts of the parasite [15]. The number of network edges mirrored depth-specific trends in relative abundance among putative hosts (Figs 1A and 2C). For example, the number of edges between Syndiniales-Arthropoda and Syndiniales-Dinophyceae was highest in the photic zone, while associations with Polycystinea, Acantharea, and RAD-B were elevated in the aphotic zone (Fig. 2C). The exception were associations between Syndiniales and RAD-B that were most prevalent among radiolarians in deeper waters, despite RAD-B accounting for <10% of sequence reads (Figs 1A and 2C). Positive and negative edges for each respective pairing were tightly linked with depth (Fig. 2C), with some exceptions (e.g., increase in negative Syndiniales-Arthropoda edges at 40–160 m). Interpreting the sign of network edges is difficult without further experimental context [59]. Positive edges may indicate infection (copresence), while negative edges may represent lysis that separates groups (mutual exclusion); however, edges can also imply an overlapping or different ecological niche.

Dinoflagellates are the most well-studied hosts of Syndiniales [60], with infection reported in culture and in the field, including in genera that were common at BATS, such as *Gyrodinium*, *Gymnodinium*, and *Prorocentrum* (Supplementary Fig. S8; Table S3). Though poorly understood, parasite spores may also represent a potential food source for heterotrophic or mixotrophic protists [61]. Relationships between Syndiniales and other hosts, such as copepods and radiolarians, are even less resolved. Copepods, which in our networks mostly involved *Calocalanus*, *Clausocalanus*, *and Triconia* (Supplementary Fig. S8; Table S3), may be directly infected by Syndiniales Groups I and IV [62], though it is more likely copepods are associated with parasites via uptake of infected prey [63]. Direct infection has not yet been observed in radiolarians. However, many have speculated on a parasitic relationship [15, 37, 64], given their shared ecological niche in deep waters and the presence of Syndiniales sequences in single-cell radiolarian isolates [65, 66]. Our network analysis supports the role of radiolarians as potentially important hosts of Syndiniales in the aphotic zone. We identified several putative parasite–host relationships among radiolarians (Supplementary Fig. S8; Table S3), involving Group I (Clade 2) and II (Clades 6 and 7) parasites correlated to Polycystinea (*Cladococcus* and *Heliosphaera*), Acantharea (Acantharea Group I, *Acanthoplegma*, and *Litholophus*), and RAD-B (Groups Ia, Ib, II, and III). It is also possible that radiolarians house protist symbionts that are themselves infected by spores, though such complex relationships have yet to be explored. Given the global abundance of radiolarians and their contribution to carbon export [67], it will be critical to further explore parasitic infection in this group.

In general, future work to enhance our understanding of Syndiniales population dynamics and biological relationships will hinge on integrating metabarcoding and other omics methods (metabolomics, transcriptomics, and proteomics) with quantitative approaches [43, 56, 68]. For instance, methods such as microscopy and flow cytometry can be used for cell counts and to monitor the percent of infected hosts, as parasites emit natural green autofluorescence under blue-violet excitation [69, 70]. Developing probes to target Syndiniales with quantitative PCR or digital droplet PCR should also be explored, allowing researchers to monitor and quantify parasites in the environment. Other microscopy-based methods, such as fluorescence in situ hybridization (FISH) and its improved version with catalyzed reporter deposition (CARD-FISH), have already been used to target both free-living spores and parasites within infected host cells [30, 56, 71]. CARD-FISH, along with single-cell genomics, may be especially important to verify putative hosts inferred from co-occurrence networks [72]. Ultimately, efforts to improve holistic sampling of parasite and host communities, both at scale in the field and in culture, will help to constrain parasitism within food web and ecosystem models.

### Role of Syndiniales in POC flux and vertical transport

**Figure 3:**
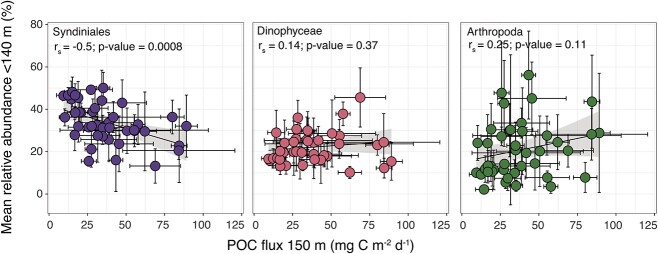
Group-specific relative abundances vs. POC flux at 150 m. Spearman rank correlations (*r_s_*) between Syndiniales, Arthropoda, or Dinophyceae mean relative abundance in the photic zone (<140 m) vs. mean bulk POC flux estimated from sediment traps deployed at 150 m. Regression line and 95% confidence intervals are shown.

Sediment trap data collected at BATS were included in our analysis to explore links between POC flux (at 150 m) and integrated Syndiniales relative abundance in the photic zone (1–140 m). Bulk POC flux at 150 m ranged from 9.4 to 89.1 mg C m^−2^ day^−1^ over the 4-year dataset (Table S2). During this time, minimal seasonality was observed, except for significantly lower mean POC flux in the fall (22.6 mg C m^−2^ day^−1^) compared with other seasons (40.5–46 mg C m^−2^ day^−1^). Previous work has reported higher POC flux in spring vs. fall at sediment traps at 150, 200, and 300 m, resulting from increased phytodetrital and fecal matter aggregation in response to elevated primary production [20, 32]. We observed a significant negative correlation (Spearman *r_s_* = −0.51; *P*-value = 0.001) between bulk POC flux at 150 m and mean Syndiniales relative abundance in the photic zone (Fig. 3), a trend that was not significant for other groups with high relative abundance (Dinophyceae or Arthropoda). In addition to POC flux at 150 m, Syndiniales relative abundance was significantly (and negatively) correlated with TOC and POC concentration (Spearman *r_s_* = −0.4 and −0.32; *P*-value < 0.001). Parasites are thought to have a similar biogeochemical impact as viruses, rerouting carbon away from POC and into pools of labile dissolved and particulate organic matter (DOM/POM) that fuel bacterial production [28]. Our findings support the expected ecological role of protist parasites in the ocean [26] and suggest that Syndiniales, in particular, significantly drive particle flux attenuation through remineralization of host carbon. Yet, the contribution of parasitism to DOM remains unclear in microbial systems [73] and warrants deeper investigation through omics-based surveys and controlled laboratory experiments.

**Figure 4:**
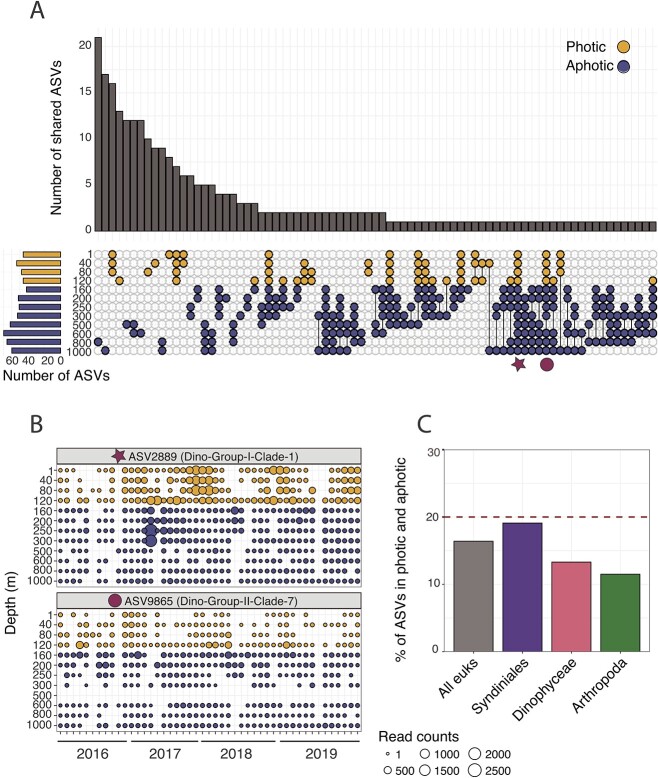
Vertical transport of Syndiniales ASVs at BATS. (**A**) UpSet plot to visualize the intersection of Syndiniales ASVs between depth networks. Horizontal bars on the left show the number of Syndiniales ASVs within each depth network. Points and lines between points indicate the intersection, while bar plots on the top panel represent the number of shared Syndiniales ASVs. Networks are referenced by their position in the photic (gold) vs. aphotic zones (blue). Overlap is based on presence–absence data. (**B**) Example profiles of specific Syndiniales ASVs from the intersection plot (red star and circle) to show spatial and temporal changes in rarefied reads counts at the ASV level. (**C**) The percentage of ASVs for all eukaryotes, Syndiniales, Dinophyceae, and Arthropoda present at any time in both the photic (1–120 m) and aphotic (160–1000 m) zones. The red dashed line indicates upper limit estimates of percent of primary production exported from photic zone each year [74].

Our results also suggest that a proportion of Syndiniales are exported out of the surface, based on the presence of Syndiniales ASVs in both the photic and aphotic zones (Fig. 4A–C). Indeed, Syndiniales ASVs are consistently reported within sediment traps across diverse marine systems [18, 19, 37]. While our work cannot conclude the exact mechanisms of export, Syndiniales may be contained within sinking phytodetritus, fecal aggregates, or fecal pellets [10], all of which constitute the bulk of sinking POC at BATS [20]. Infected hosts may be transported to deep waters via zooplankton fecal matter, expedited through diel migration [75]. Though infection is rapid (2–3 days), motile parasite spores can survive outside the host for up to 15 days under laboratory conditions [25, 27]. Therefore, it may be possible for spores to survive on sinking aggregates, which at BATS range in size from 60 to 1862 μm [20, 76]. There is also evidence that Syndiniales become more abundant on particles over time, indicating active infection of attached hosts [10]. Spores may survive without their preferred hosts by widening their host range, becoming dormant inside host cysts [69], or acquiring alternative energy sources (e.g., uptake of DOM). Osmotrophy of DOM has been shown in haptophytes [77], which warrants its investigation in other protist groups such as Syndiniales that may possibly utilize DOM to survive under host-limited conditions. Lastly, Syndiniales may reach deep waters through convective mixing [42, 78], either directly or on particles.

Though negatively linked with POC flux at 150 m (Fig. 3), 19% of Syndiniales ASVs showed signs of vertical transport at BATS (Fig. 4A–C). Compared with Syndiniales, fewer ASVs (12–16%) were present throughout the water column for Arthropoda, Dinophyceae, and all eukaryotes (Fig. 4C), suggesting less vertical connection. Moreover, Syndiniales ASVs in the networks were present across seasons (Supplementary Fig. S9), with signals of vertical transport consistent over the 4-year period and seasonally recurrent (Fig. 4B). As demonstrated here and hinted by others [15, 26], Syndiniales parasites likely play an important role in modulating sinking POC and may serve as biomarkers for carbon export. As an example, the percent of sinking parasite ASVs at BATS is concurrent with the percent of primary production (10–20%) sinking each year from the photic zone [74]. While not directly comparable and requiring additional evidence, our results suggest the potential application of molecular tools to capture export by evaluating Syndiniales ASVs and their correlation to analogous flux estimates. Given the dominance of Syndiniales relative abundance in the surface ocean, at depth, and in sediment traps, we propose using Syndiniales parasites as a biomarker for carbon export flux in the ocean. Additional field and laboratory work is needed to characterize sinking mechanisms, partition infected hosts vs. spores with sample fractionation [44], and incorporate omics with quantitative methods to better resolve parasite–host dynamics and the impact of parasitism on ocean biogeochemistry.

## Conclusions

The influence of parasitism on marine microbial food webs and carbon cycling is unclear. This study provides detailed information on depth-specific population dynamics and trophic relationships among the widespread protist parasite group, Syndiniales, and links parasites with POC flux and vertical transport of sinking POC in the oligotrophic North Atlantic Ocean. Syndiniales were connected to a range of known and putative hosts, such as dinoflagellates, copepods, and radiolarians, with associations varying with depth based on changes in host composition. A strong and negative correlation between Syndiniales in the photic zone and POC flux to 150 m implies that parasites are contributing to POC flux attenuation and carbon recycling as particles move from the photic to aphotic zones, an ecological role that has been suggested by others [26]. Syndiniales communities in the aphotic zone may represent resident clades that are adapted to specific ecological niches [15], as well as a subset of parasites that sink and may exhibit opportunistic infection strategies. Given the strong connection with POC flux from the photic zone, it may also be expected that parasites contribute to labile DOM at depth that can fuel microbial communities [48]. In total, these findings elevate the role of Syndiniales in marine food webs, highlighting their importance to species networks at all depths and potential use as biomarkers for carbon export.

## Materials and Methods

### Sample collection

Monthly seawater samples were collected at BATS over a 4-year period (February 2016 to December 2019) at 12 discrete depths in the water column (1, 40, 80, 120, 160, 200, 250, 300, 500, 600, 800, and 1000 m). At each depth and month, 4 l of seawater was filtered through 0.22-μm Sterivex filters (MERCK, MA, USA) and filters were stored at −80°C [33]. Only a single replicate filter was collected. Each profile was retrospectively assigned to one of four seasons [33] that corresponded to the position of the mixed layer depth (e.g., mixed in winter vs. stratified in summer). CTD profiles (temperature, oxygen, and salinity) and discrete chemical and biological data were also collected each month and considered here to relate to Syndiniales read counts. Dissolved nutrients (NO_3_, NO_2_, PO_4_, and SiO_4_), TOC, total *p*CO_2_, particulate organic carbon and nitrogen (POC and PON), total alkalinity, total nitrogen, and bacteria cell density were included. Details on sample collection and processing for these measurements are available at https://bats.bios.edu/data/. Values for all environmental variables are in Table S2. To explore depth-related shifts in biology and carbon export, we partitioned samples based on their position in photic vs. aphotic zones. The photic zone is well established and operationally defined at BATS as ~1–140 m (to the 0.1% PAR), transitioning to the aphotic zone (160–1000 m) below 0.1% PAR [40, 78].

Bulk POC flux was estimated from monthly surface-tethered Particle Interceptor Traps that were deployed at 150, 200, and 300 m and collected particles for 3 days [20]. Triplicate trap tubes were fitted with acid-cleaned 0.8-μm polycarbonate filters at the bottom and filled with poisoned seawater brine. Trap filters were processed based on standard BATS protocol [79] to determine bulk POC flux using C/N analyses [20]. Mean Syndiniales relative abundance in the photic zone (1–140 m) was correlated with bulk POC flux at 150 m via Spearman rank (*r_s_*) correlation. Similar correlations were made with other important eukaryotic groups in the photic zone, such as Dinophyceae and Arthropoda. Though deployed at the same site, there was a ~2–3 days lag between DNA collection and sediment trap recovery. Bulk POC flux data at 150 m are also included in Table S2.

### DNA extraction, PCR, and 18S rRNA gene metabarcoding

DNA extraction from Sterivex filters has been described in [33]. Primers were used to amplify the 18S V4 hypervariable region: 5-CCAGCA [GC]C[CT]GCGGTAATTCC-3 and 5-ACTTTCGTTCTTGAT[CT][AG]A-3 [80]. Initial PCR runs consisted of a denaturation step at 98°C for 30 s, 10 cycles at 98°C for 10 s, 53°C for 30 s, and 72°C for 30 s, followed by 15 cycles at 98°C for 10 s, 48°C for 30 s, and 72°C for 30 s, and a final elongation at 72°C for 10 min [33]. PCR reactions (25 μl) were run with 2 μl of target DNA, 1 μl of each primer, and 12.5 μl of KAPA HiFi HotStart ReadyMix (Kapa Biosystems; Wilmington, MA, USA). A second PCR was performed using dual Illumina indices as per the Illumina Nextera XT Index Kit. Amplicon sequencing was carried out on a MiSeq (2 × 250 bp) at the Center for Genome Research and Biocomputing at Oregon State University.

Primers were removed from demultiplexed reads using Cutadapt [81]. ASVs were assigned to trimmed 18S reads using paired-end DADA2 in QIIME 2 [82]. Taxonomy was assigned using a Naïve Bayes classifier trained with the Protistan Ribosomal Reference (or PR2) database (Version 5.0.1; [83]) and trimmed to the primer region [84]. The current PR2 release included several updates, changing kingdom to domain and adding a new subdivision level for a total of 9 taxonomic levels [85]. The PR2 database includes some metazoan assignments, considered here to explore potential Syndiniales-zooplankton relationships [62]. Resulting taxonomy, ASV count, and metadata files were imported into R (Version 4.2.1) using qiime2R (https://github.com/jbisanz/qiime2R) and merged with phyloseq [86]. Groups assigned to Craniata (28 ASVs), Streptophyta (183 ASVs), Rhodophyta (1 ASV), Bacteria (55 ASVs), Opisthokonta_X (12 ASVs), and unassigned reads at the class level (8098 ASVs) were filtered out of the dataset. Samples with less than 5000 read counts (3 samples) and global singletons (ASVs present once; 3257 ASVs) were also removed. Following these filtration steps, the 4-year 18S dataset at BATS yielded 67 170 sequence reads on average (15 063–206 637) from 500 samples, mapping to 48 786 ASVs. Species accumulation curves were constructed using the R package ranacapa and a step size of 100 [87]. The number of 18S reads vs. ASVs was saturated across all samples and depths, indicating that sufficient sequencing depth was reached (Supplementary Fig. S10). Count tables were rarefied to the minimum read count (15 063 reads). The filtered ASV taxonomy and read counts table is provided in Table S4.

Changes in mean relative abundance with depth and season were observed using stacked bar plots in the R package microeco [88]. Taxonomy was visualized at the class level, as well as order and family (clade) level within Syndiniales. Subsequently, phyloseq objects were trimmed to include only Syndiniales ASVs to focus on their population dynamics. Prior to running an ordination, we tested if homogenous dispersion existed among depths and seasons using the betadisper function in vegan [89]. These tests were significant, indicating that species composition was influenced by dispersion and not solely by the location of centroids. Spatial and temporal trends in Syndiniales composition were observed via principial coordinates analysis of a Bray–Curtis dissimilarity matrix and tested for significance with permutational multivariate analysis of variance (PERMANOVA) using the adonis2 function in vegan with 9999 permutations [89]. Pairwise comparisons (with Bonferroni correction) were made using the R package pairwiseAdonis [90] to test for differences between sampling depths or seasons (9999 permutations). Mean Bray–Curtis dissimilarity was compared between discrete depths with one-way Analysis of Variance in microeco [88]. Mean Shannon diversity index was estimated for samples in the photic vs. aphotic zones using the estimate_richness function in phyloseq [86].

### Partial least squares regression

The influence of environmental (predictor) variables on Syndiniales read counts (response variable) was determined with PLSR using the R package mdatools [91]. An initial PLSR model was generated using rarefied Syndiniales read counts. Predictor and response variables were centered and scaled (standardized) when performing the core pls function in mdatools. Outliers were detected using a Data Driven robust method [92] and removed (Supplementary Fig. S5). After running a final PLSR model, Root Mean Squared Error plots were used to select the optimal number of components (Supplementary Fig. S5). In this case, four components were optimal. Regression coefficients and variable influence on the projection (VIP) scores were estimated for each predictor variable (Supplementary Fig. S5). VIP scores >1 are considered most important to the model [91]. Spearman rank (*r_s_*) correlations were used to further explore predictor-response effects.

### Network analysis

Network analysis was applied to observe associations between Syndiniales and potential host organisms throughout the water column. Twelve separate networks were constructed, one for each depth, using the SParse InversE Covariance estimation for Ecological Associations and Statistical Inference (SPIEC-EASI) package in R (Version 1.1.0; [54]). SPIEC-EASI aims to minimize spurious edges from compositional data and infer direct associations between ASVs [54]. To minimize dense networks, 18S datasets were subsampled to include only the top 150 most abundant ASVs at each depth. The top 150 ASVs closely represented the full community at each depth (Supplementary Fig. S6). ASV count tables were centered log-ratio (clr) transformed and networks were run using the Meinshausen–Buhlmann’s neighborhood selection method and an optimal sparsity threshold of 0.05 [93]. Depth networks were filtered to include only positive and negative edges between Syndiniales ASVs and other 18S groups. Networks were visualized in Cytoscape [94]. Mean degree or the number of edges connected to a given ASV [51] was estimated for Syndiniales and potential host ASVs to indicate the specificity of connections.

To observe the overlap of Syndiniales ASVs between depths and seasons, we constructed UpSet plots using the R package ComplexUpset [95] that were based on presence–absence data. We further estimated the percent of Syndiniales ASVs that were present in the photic and aphotic zones, which may indicate vertical transport of parasites in the water column. Percent of transported parasites were compared with ASVs from other major taxonomic groups in the photic zone (Arthropoda and Dinophyceae), as well as all eukaryotic ASVs.

## Supplementary Material

ISME_cleaned_supp_ycae014

Table_S1_ycae014

Table_S2_ycae014

Table_S3_ycae014

Table_S4_ycae014

## Data Availability

All code and files needed to reproduce results from this study are available at https://github.com/sra34/BATS-parasites. The GitHub repository also includes metadata, network files, and raw QIIME 2 files needed for the analysis; 18S rRNA sequences have been deposited in the Sequence Read Archive under BioProject PRJNA769790.
